# Quantitative structure–activity relationship model to predict the stability constant of uranium coordination complexes for novel uranium adsorbent design†

**DOI:** 10.1039/d5ra02220g

**Published:** 2025-05-19

**Authors:** Hyun Kil Shin, Youngho Sihn

**Affiliations:** a Prediction Model Research Center, Korea Institute of Toxicology Daejeon 34114 Republic of Korea hyunkil.shin@kitox.re.kr; b Human and Environmental Toxicology, University of Science and Technology Daejeon 34113 Republic of Korea; c Korea Atomic Energy Research Institute Daejeon Republic of Korea

## Abstract

A quantitative structure–activity relationship (QSAR) model for predicting the stability constant of uranium coordination complexes to accelerate the discovery of novel uranium adsorbents was developed and evaluated. Effective uranium adsorbents are crucial for mitigating environmental and health risks associated with uranium wastewater, an unavoidable byproduct of nuclear fuel production and power generation, as well as for sequestering uranium from seawater. QSAR modeling addresses the limitations of quantum mechanics calculations and offers a time- and cost-efficient computational approach for exploring vast chemical spaces. The QSAR model was built using a dataset of 108 uranium complexes, incorporating features such as physicochemical properties, coordination numbers of ligands, molecular charge, and the number of water molecules. Catboost regressor achieved an *R*^2^ of 0.75 on the external test set after hyperparameter optimization. Applicability domain analysis was conducted to evaluate model predictive performance. The QSAR model predicts stability constants from the molecular composition alone and is a valuable tool for the efficient design of safer and more sustainable uranium adsorption materials, potentially improving uranium collection processes.

## Abstract 2

QSAR model was developed for uranium adsorbent design.

## Graphical abstract

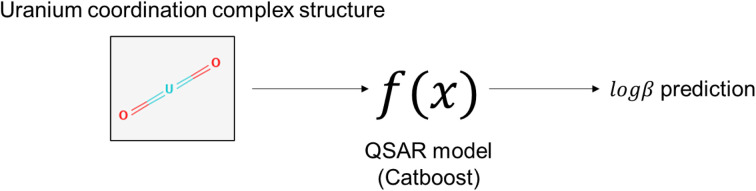

## Introduction

Uranium plays an indispensable role in meeting the world's growing energy demands, specifically in the production of nuclear energy. However, terrestrial uranium resources are finite, and as high-grade ores become increasingly scarce, the need for alternative sources of uranium is becoming more urgent. One such alternative is the extraction of uranium from seawater. The world's oceans contain approximately 4.5 billion tons of uranium, enough to meet the world's uranium demand for over 10 000 years.^1^ This immense resource, however, is distributed at a very low concentration of about 3.3 ppb,^2^ making its extraction technically challenging. The retrieval of uranium is also associated with safety concerns since the traditional methods of uranium extraction, primarily terrestrial mining, pose significant environmental and health risks. Uranium mining and milling generate substantial radioactive waste, leading to contamination of water, soil, and air, with long-term consequences for ecosystems and human health. Thus, effective treatment methods are needed for the safe and sustainable extraction and use of uranium.^3^ Uranium wastewater containing uranyl ions poses direct environmental and health hazards. With uranium wastewater being an unavoidable byproduct of nuclear fuel production and power generation, proper waste management is imperative.

While adsorption is an effective method for sequestering uranium from wastewater,^4^ the development of adsorbent materials that can efficiently capture dilute concentrations of uranium present in seawater, is a more difficult challenge. These materials must exhibit high selectivity for uranium over other metal ions, be resistant to biofouling, and maintain their performance over multiple adsorption–desorption cycles.^5^ The extraction of uranium from seawater has been explored for decades, but most of the adsorbents were ineffective except polymeric adsorbents^6^ and amidoxime-based materials.^7^ Amidoxime-based polymers have emerged as the leading material for uranium adsorbents due to their strong affinity for the uranyl ion (UO_2_^2+^). The amidoxime functional group, which forms stable complexes with uranium, is central to the ability of amidoxime-based adsorbents to selectively adsorb uranium from seawater. However, the large-scale implementation of seawater uranium extraction remains limited by the high costs associated with the low efficiency of uranium extraction. Therefore, new adsorbents which can efficiently extract uranium from seawater must be continuously explored and developed.

The adsorption performance of uranium adsorbents can be assessed through the stability constant, which indicates the strength of the interaction between adsorbent material and uranium to form complexes.^8^ The stability constant is represented as follows:1
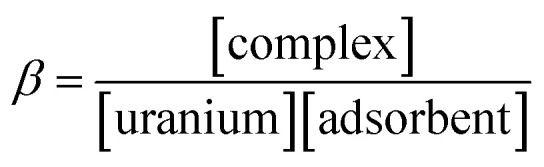
where *β* is the stability constant, and [complex] is the concentration of the uranium-adsorbent coordination complex, and [uranium] and [adsorbent] are the concentrations of each species. Computational methods can be used to determine *β*. Quantum mechanics (QM) calculations can be used to predict *β*,^9^ and QM methods have been used in material design often. However, QM methods cannot explore the vast chemical space quickly and cost-effectively. Consequently, the use of machine learning (ML) models for novel material design is more efficient.^10^

The quantitative structure–activity relationship (QSAR) model is an ML model whose input is a representation of the molecular structure and whose output is the activity of the input molecule (*i.e.*, experimentally measured properties). In the QSAR model, features calculated from molecular structures (also called descriptors), are used to predict the activity variation of the molecule as a consequence of structural variation. To the best of our knowledge, prior to this work, only one QSAR model for *β* prediction has been reported. Zahariev *et al.* developed a QSAR model based on graph neural network models and traditional ML models for predicting *β* for metal–ligand complexes with the aim of designing new selective ligands for the target metal ion.^11^ The predictive accuracy of the QSAR model is heavily dependent on the applicability domain (AD) of the model.^12^ The predicted result is reliable when the input molecule has similar structural features with training data. If the specific target molecules only take small portion in the entire training set, the structural pattern of such molecules are not well trained; therefore, there is a high possibility that the model can't produce reliable prediction results on the molecule. Thus, for novel uranium adsorbent development, the developed model focuses on the chemical space of uranium complexes can provide better and reliable prediction results than the general model.

In this study, we developed a QSAR model to predict *β* for uranium complexes. In total, 108 uranium complexes were collected with their stability constants. Descriptors used in the model are the physicochemical properties, coordination numbers according to ligand atom, charge number, and the number of water molecules due to hydroxylation. The molecular formula of the uranium complexes was used to calculate four physicochemical properties, specifically, water solubility, boiling point, melting point, and pyrolysis point using a neural network model for inorganic compounds. Catboost achieved the best prediction performance in the external test set (*R*^2^: 0.75). Therefore, the evaluation results confirm that the model built in this study is capable of discovering novel uranium adsorbents.

## Materials and methods

### Data preparation

The stability constant (log *β*) was collected from OECD-NEA thermochemical database and research articles with the structure information of uranium coordination complexes (ESI†). In data collection, we compared the different log *β* values from different research articles and selected values with no significant discrepancies. However, data obtained from OECD-NEA thermochemical database, a widely accepted and rigorously evaluated dataset, were used even if single value was available. The log *β* values in our dataset are considered representative, reducing concerns over variability. The molecular formula and ligand atoms were used to represent the molecular structure of uranium coordination complexes. The total data size was 108, and the data set was divided into training and test sets with a ratio of 8 : 2; thus, the training set comprised 86 data points and the test set comprised 22 data points.

### Feature preparation

Features were prepared based on the structural properties of the uranium coordination complexes such as the coordination number of each ligand (ligand_N, ligand_O, ligand_F, and ligand_Cl), charge, number of water molecules through hydroxylation (H_2_O), molecular weight (MW), and four physicochemical properties: aqueous solubility (log *S*), melting point (mp), boiling point (bp), and pyrolysis point (pp). These four physicochemical properties were predicted based on the molecular formula of the uranium coordination complexes using a neural network model.^13^ The physicochemical property prediction models used here were developed for inorganic compounds, in contrast to most existing models focusing on organic molecules. These models use the electron configuration of inorganic molecules based on the composition to calculate the four physicochemical properties. In the collected dataset, the coordination complexes have four different ligand atoms such as N, O, F, and Cl. Thus, the number of ligand atoms was used as a feature. Prepared features are available in ESI tables (training set: Table S1, and external test set: Table S2†).

### Machine learning model development

**Fig. 1:**
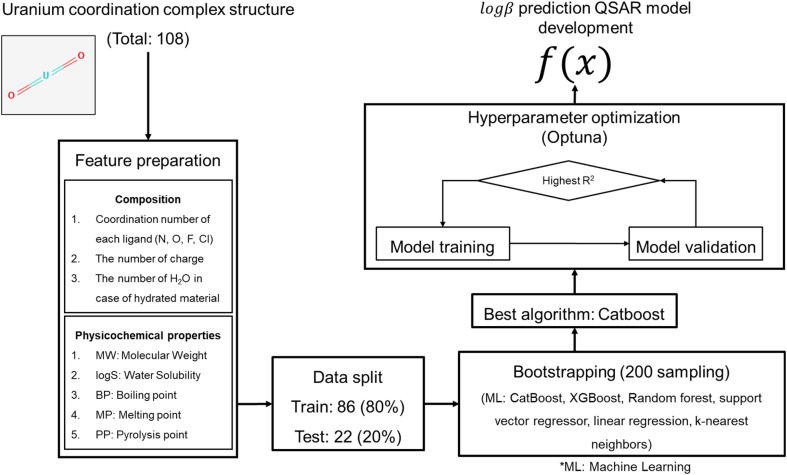
QSAR model development process. The composition of the molecule was used in feature preparation and physicochemical properties were calculated based on molecular formular of the compounds. The data set was randomly split by 8 : 2 (training : test). The best machine learning algorithm was selected after bootstrapping (200 sampling) and was further developed through hyperparameter optimization.

QSAR models were developed according to OECD QSAR validation guideline (Fig. 1).^12^ Two ML algorithms were tested in model development: extreme gradient boosting (XGBoost), and support vector regressor (SVR). Particularly, XGBoost and SVR were used in this study since these two ML algorithms showed good prediction accuracy compared to graph neural network in few regression tasks.^14^ Given that the data size is small in this study, deep learning algorithms can't be applied; thus, two ML algorithms were tested in this study. Bootstrapping was used for internal validation instead of *n*-fold cross validation due to small size of dataset. Bootstrapping allows duplication when sampling data. Sampled data chunk is used as training set, and unsampled data points are used as validation set. Recommended sampling round is 20 to 200. In this study, each ML algorithms were evaluated with 200 rounds of bootstrapping. The optimum set of hyperparameters were searched using Optuna library version 3.4.0.^15^ ML models were developed with scikit-learn (version 1.3.0),^16^ xgboost library (version 1.7.6),^17^ and catboost library (version 1.2.7). The model performance was evaluated using the root mean squared error (RMSE) and *r* square (*R*^2^). The model developed in this work was used to predict the *β* of candidate materials.

Model was further validated through y-randomization test^18^ to check if the model's performance is achieved by coincidence or not. In the test, the model was trained on the randomized endpoint, and compare the model's performance between original endpoint and shuffled endpoint. *Z*-score was calculated as
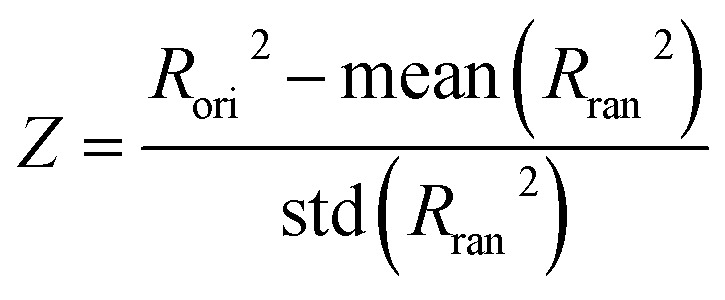
where *R*_ori_^2^ is *R*^2^ of the model from original dataset, and *R*_ran_2 is *R*^2^ of the model from shuffled endpoint. During y-randomization, endpoint was shuffled 30 times. *R*_ran_^2^ was calculated after each shuffling, and average and standard deviation of *R*_ran_2 were calculated for *Z*-score calculation. The model's performance was based on strong correlation between features and endpoint if *Z*-score is over 3.

### Applicability domain analysis

The QSAR model makes a valid prediction when the input molecule is sufficiently similar to the training set. Thus, it is crucial to perform AD analysis to determine if the prediction is valid. Basically, AD analysis is an outlier detection step. The simplest way to identify outliers involves checking the feature range. Any data whose feature value is out of the training set's feature value range is considered an outlier. The outlier was identified using the leverage and warning values. The leverage value was calculated for the *i*-th compound (*h*_*i*_) with the warning value for the model as below:

2 *h*_*i*_ = *x*_*i*_(*X*^*T*^*X*)^−1^*x*_*i*_^*T*^

3
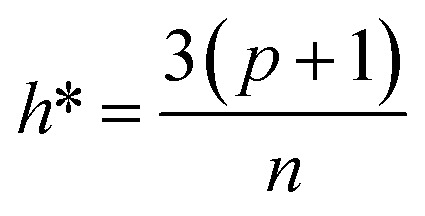
where *X* is the feature matrix of the training set, *x*_*i*_ is a feature vector of *i*-th compound, *p* is the number of features used in the model, *n* is the number of compounds in the training set, and h* is a warning value. If *h*_*i*_ is larger than h*, then the compound is considered as an outlier. Then, the leverage value of the model was examined, and the outlier was identified using a William plot from the training and test sets. In candidate material prediction, the leverage value was compared with the warning leverage to check for outliers. Moreover, all the feature value ranges were examined, and the candidate material was considered an outlier if one of the features has a value outside the range of the values in training set.

## Results

### Dataset analysis

**Fig. 2:**
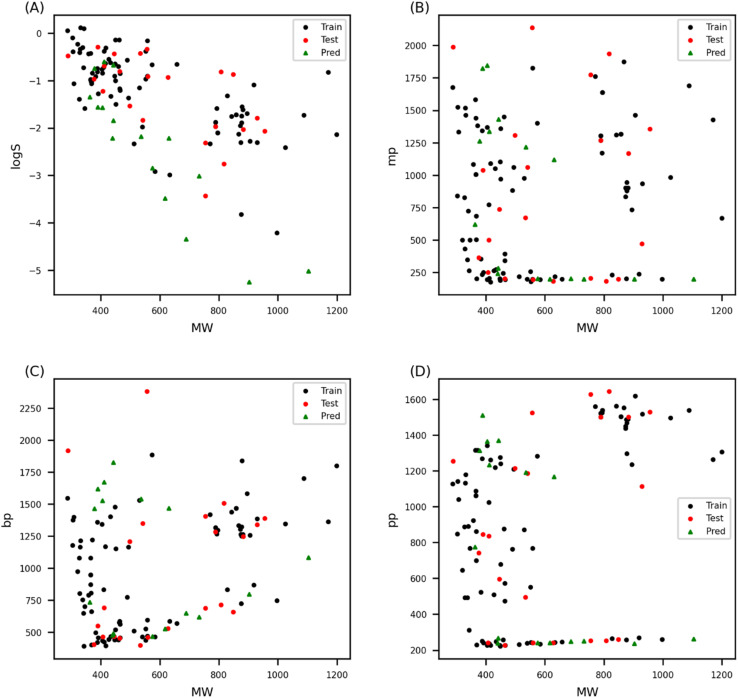
Chemical space of uranium coordination complexes visualized with molecular weight against aqueous solubility (A), melting point (B), boiling point (C), and pyrolysis point (D). The chemical space shows that training set and test set have similar structural patterns. Moreover, candidate materials also show similar chemical spaces, confirming that the model developed with training data and validated with test data can make reliable prediction on the candidate material.

Chemical diversity of dataset was examined through chemical space in Fig. 2. The range of molecular weight was from 287 to 1199, which indicated that the size of molecules was highly varied in the dataset. Also, physicochemical features (log *S*, mp, bp, and pp) were calculated base on the composition of the molecules. Wide range of each features showed that the molecular composition was also diverse. Molecular formular of the molecules is available in Table S1 (training set) and Table S2 (test set).† Training and test data split is important issue since the model's performance may not be correctly evaluated according to the data distribution in training and test set. Data was randomly split multiple times to test if random sampling may cause discrepancy in data distribution between training and test set; however, test set was never biased. Therefore, training and test data was prepared with random split. Chemical space analysis showed that training and test sets were well diversified, which indicated that the model performance evaluated by external test set can be trusted within AD of the model (Fig. 2).

The QSAR model prediction reliability was evaluated *via* AD analysis. This involves checking the chemical space which represents the structural diversity of the compounds in each dataset. Normally, the chemical space is visualized based on the molecular weight and the water/octanol partition coefficient (log *P*) for organic molecules; however, the log *P* model does not apply to inorganic molecules. In this study, four physicochemical properties were calculated from the molecular formulas of the uranium coordination complexes; therefore, the chemical space of the data set was compared based on the molecular weight and the four physicochemical properties. The chemical spaces of the training set, test set, and candidate materials were compared as shown in Fig. 2. The training and test sets show a similar distribution in the chemical space. Also, the chemical space of the candidate material was similar to the training and test data sets; therefore, we can conclude that the model trained and validated with the dataset can be reliably used to predict the *β* of the candidate materials.

### Model development

**Fig. 3:**
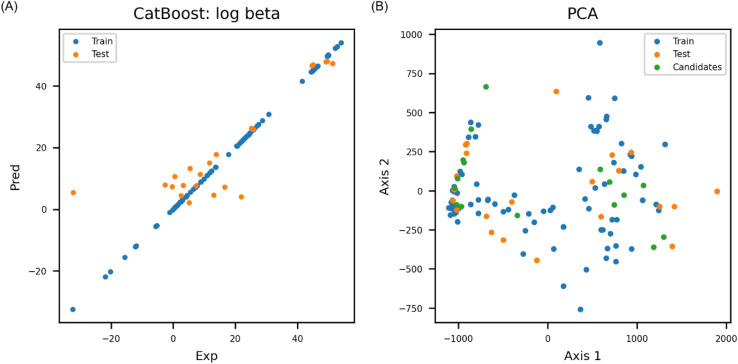
(A) Parity plot of catboost regression model (RMSE: 10.28 and *R*^2^: 0.75 on external test set). (B) PCA showed that chemical space covered by training and test set well overlapped with candidate absorbents (explained variance of the first axis: 0.84).

According to the internal validation result, catboost achieved highest prediction accuracy compared to other ML models (Table 1). The catboost model exhibited *R*^2^ of 0.75 (Fig. 3A) and RMSE of 10.28 for external test set (Table 2). PCA was conducted to check if chemical space of candidate molecules were well covered by the training and test set (Fig. 3B). Explained variance of the first axis in PCA being 0.84 indicated that the candidate molecules resided within the chemical space of the final model. Y-randomization test was also conducted to check if the model's prediction accuracy was merely by chance. Y-randomization test was performed three times, and the lowest *Z*-score among the Y-randomization test was 4.48, which showed that the model's performance was based on good correlation between feature and endpoint, and it wasn't achieved by mere coincidence.

**Table 1:** Bootstrapping validation results

Abb.	Model	*Q* ^2^ (bootstrapping^a^)	
		Mean	Std^b^
Catboost	Catboost regressor	0.70	0.10
XGBoost	Extreme gradient boosting	0.62	0.17
RF	Random forest	0.61	0.16
kNN	*K*-Nearest neighbors regressor	0.56	0.19
LR	Linear regressor	0.56	0.17
SVR	Support vector regressor	0.23	0.10

a 200 times sampling was applied in the internal validation.

b Standard deviation.

**Table 2:** Best model performance

Catboost	*R* ^2^	RMSE	NRMSE	Endpoint range
Train	0.99	0.04	0.05%	86.4
External validation	0.75	10.28	12.31%	83.5

Feature importance was analyzed by developing models with composition features, and physicochemical properties alone. When catboost was evaluated with five physicochemical properties (MW, log *S*, bp, mp, and pp) alone, the model only achieved 0.6 for *Q*^2^ in bootstrapping (200 round sampling). With the six composition features alone (*i.e.*, ligand_N, ligand_O, ligand_F, ligand_Cl, charge, and H_2_O), the model achieved *Q*^2^ 0.68. The composition features have low variance; therefore, there is a clear limitation to represent structural diversity of uranium coordination complex with the composition features alone. According to the experiment, composition features and the physicochemical properties both were needed to represent molecular structure of uranium coordination complex; thus, the model performed best when all the features were used together (Table 3).

**Table 3:** Feature importance analysis

Catboost	*Q* ^2^ (bootstrapping^a^)	
	Mean	Std^b^
PhyChem^c^ alone	0.60	0.12
Composition^d^ alone	0.68	0.11
PhysChem & composition	0.70	0.10

a 200 times sampling of bootstrapping.

b Standard deviation.

c PhysChem: physicochemical properties (molecular weight, water solubility, melting point, boiling point, pyrolysis point).

d Composition: coordination number of ligand (N, O, F, Cl), charge, and the number of water molecules.

The charge state of the uranium complex significantly impacts *β*. Higher charges typically result in stronger electrostatic interactions between the uranium ion and the ligands, leading to more stable complexes. This is consistent with the observed feature importance, where the inclusion of molecular charge as a feature notably improved the model performance. The coordination number, which reflects the number of ligand atoms bonded to the central uranium ion, directly influences the stability of the complex. Ligands with a high coordination number can donate more electrons to the uranium ion, stabilizing the complex through stronger bonding interactions. The sensitivity of the model to changes in coordination number underscores its critical role in determining complex stability. Additionally, the number of water molecules in the system is significant, as hydration can either stabilize or destabilize the complex depending on specific interactions with the central metal ion and surrounding ligands. These composition features, when incorporated into the model, allow for a more comprehensive representation of the physicochemical factors governing complex stability, thus explaining the significant improvement in model performance.

### Prediction result analysis

**Fig. 4:**
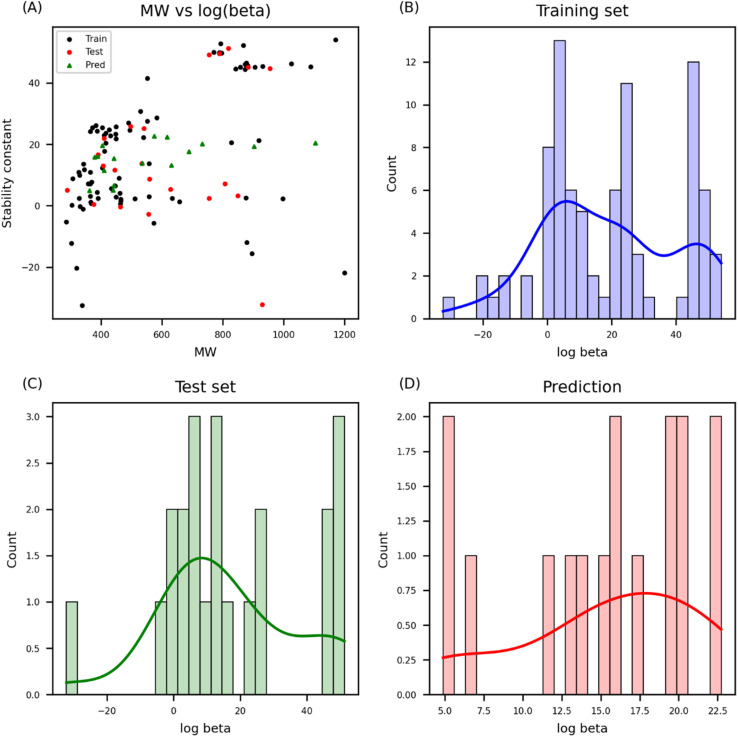
Comparison of the experimental *β* and the predicted *β*. The experimental *β* from the training and test sets is visualized with the predicted *β* from the candidate materials (A). The training set (B) and test set (C) have a wider range of *β* values whereas the predicted *β* has a narrower range (D).

**Fig. 5:**
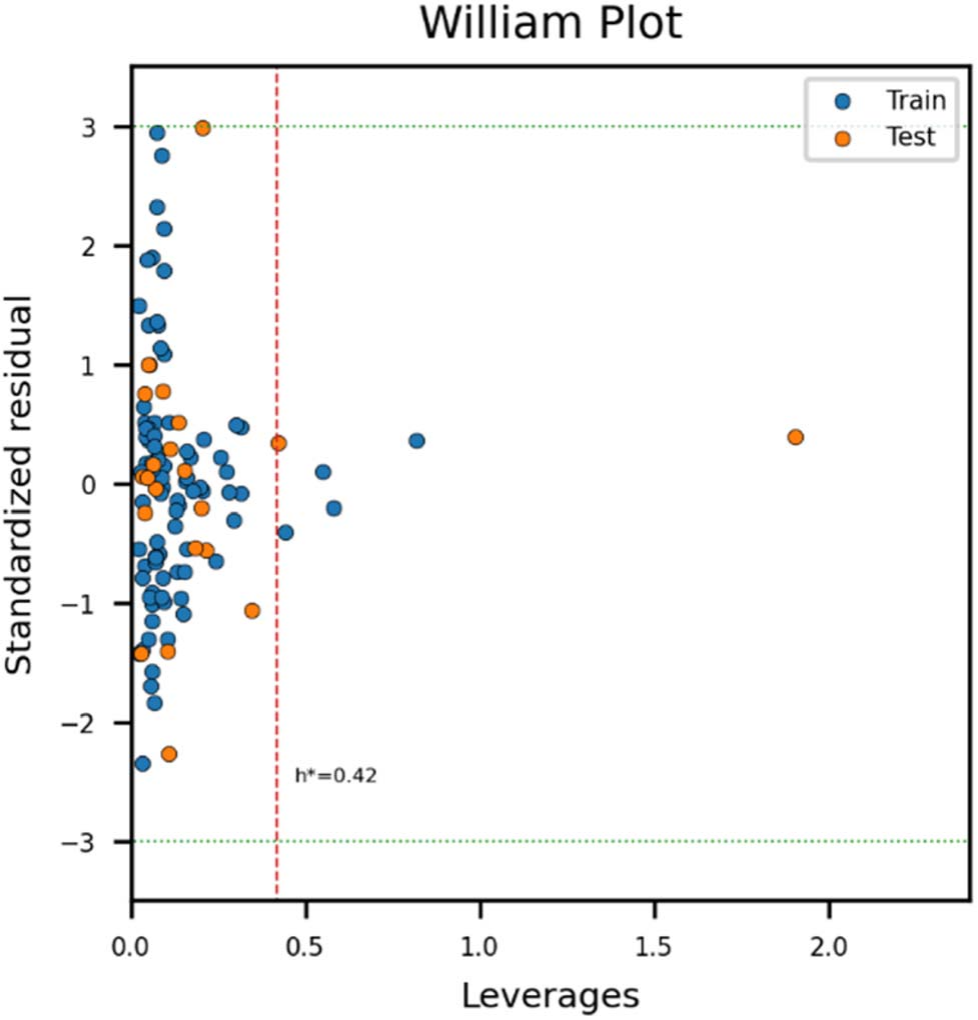
William plot for analyzing the applicability domain (AD) of the model. Only one compound was outside of the range of values of the test set, and 93% of compounds in the training set was in the AD domain (82 out of 86).

**Fig. 6:**
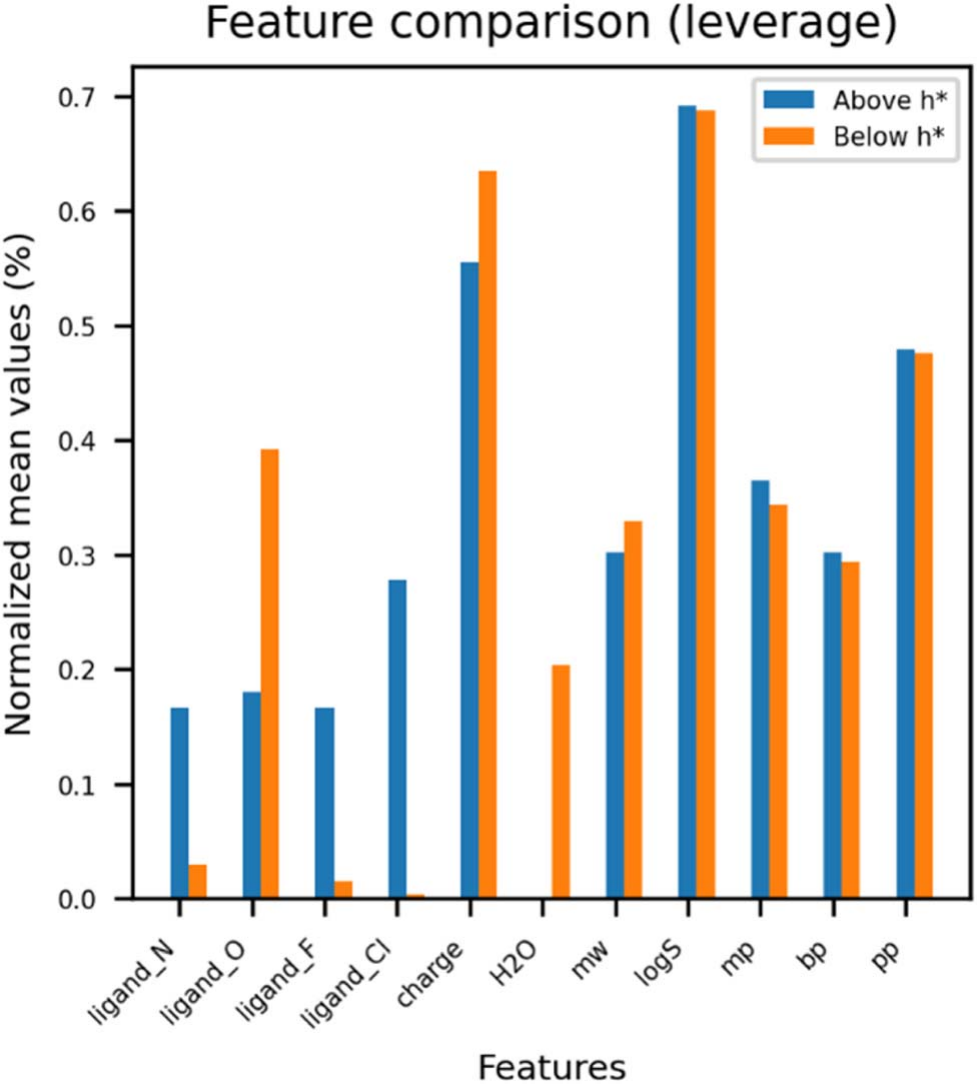
Averaged feature values were visualized between outliers and other data points. Three features such as ligand ‘O’, charge, and H_2_O have large gaps between them in common. Additionally, (A) four molecules exceeding warning values have significant discrepancies in ligands features (N, F, and Cl).

The range of prediction values was examined, as the prediction values for the uranium complex should fall between the maximum and minimum values in the training set. According to the analysis comparing experimental and predicted results, the predicted values of *β* for the candidate materials were within the range of *β* in both the training and test sets (Fig. 4). AD analysis was further performed on the training and test sets (Fig. 5). The model domain contained 93% of the training set (6 molecules were considered as outliers), and only two molecules were out of the domain in the test set. Thus, the model developed in this study covered nearly all of the uranium coordination complexes in the training and test set. To understand characteristics of outliers, normalized feature values were compared between outliers and other compounds. Outliers showed discrepancies in the number of ligand_O and H_2_O, and charge of the molecule. Apart from the three features, four molecules with high leverage values over warning leverage have significant differences in ligand features (ligand_N, ligand_F, and ligand_Cl) whereas two molecules with large standardized residuals show significantly varied distribution in physicochemical properties such as melting point (mp), boiling point (bp), and pyrolysis point (pp) (Fig. 6).

AD analysis was applied to the candidate materials. Even though the candidate materials were in the chemical spaces of the training and test sets, there is always uncertainty in the prediction values since the model was previously not exposed to the candidate material. Therefore, we checked the feature ranges of the candidate material data and compared them to the training set. Even if the molecule was found in AD according to the leverage value, we checked the range of 11 features between the candidate data and the training set. If the molecule is out of range even in a single feature, the molecule was also marked as an outlier. After leverage analysis, 9 molecules were found in AD. Two of these had features out of the range in descriptors of the training set: the coordination number of N and aquatic solubility. As a result, 7 molecules were considered as reliably predicted by the model (Table 4). Detailed AD analysis can be found in Table S3.† The model can make reliable prediction as long as the query molecule has feature values within the range of each feature provided in the training set. Therefore, any molecule having feature values exceeding the range of each feature of the model shouldn't be used to make prediction. Moreover, predicted log *β* should not exceed the range of log *β* in the training set. Table 5 shows maximum and minimum value of log *β* and each feature.

**Table 4:** AD analysis result

AD analysis	In-domain	Out-of-domain
Leverage	9	7
Feature range	11	5
Reliable prediction	7	

**Table 5:** Range of each feature and endpoint

Values	Max.	Min.
log *β*	54	−32.4
Ligand N	3	0
Ligand O	6	0
Ligand F	4	0
Ligand Cl	2	0
Charge	2	−6
H_2_O	12	0
MW	1199.157	287.034
log *S*	0.112395	−4.21574
MP	1875.558	176.3154
BP	1887.21	391.5719
PP	1619.382	222.5845

## Discussion

The study successfully developed a QSAR model for predicting the stability constant (*β*) of uranium coordination complexes, demonstrating strong predictive performance with an *R*^2^ of 0.91 in external validation. The results indicate that both physicochemical properties and compositional features play a critical role in accurately modeling uranium complex stability, reinforcing the necessity of incorporating diverse descriptors in QSAR models for inorganic systems. The model results align with the well-established principles of coordination chemistry, where increased charge enhances electrostatic interactions, stabilizing the metal–ligand complex. Likewise, the coordination number significantly influences complex stability, as ligands with higher coordination numbers facilitate stronger bonding interactions. These observations underscore the model's ability to capture fundamental chemical principles governing uranium–ligand interactions. Compared to previous QSAR models for metal–ligand stability prediction (Zahariev *et al.*, 2024), the model developed in this study provides domain-specific applicability for uranium complexes. The use of a dataset exclusively focused on uranium coordination chemistry, rather than a broad spectrum of metal–ligand complexes, ensures that predictions are more relevant for uranium-specific adsorbent development.

The identification of seven candidate materials within the model's AD suggests that ML-driven QSAR models can be a powerful tool for guiding experimental efforts in uranium adsorbent discovery. The ability to screen potential adsorbents computationally reduces the need for labor-intensive experimental screening, making the discovery process more efficient. However, experimental validation of these candidate materials is necessary to confirm their real-world adsorption efficiency and stability under marine conditions.

## Conclusions

Nuclear energy will remain part of the world's sustainable energy mix, and uranium adsorption material development is paramount for protecting human and environmental health. Safer, effective, and cost-efficient novel materials for uranium adsorbents must be continuously developed. The QSAR model for stability constant (*β*) prediction was developed in this study to accelerate novel uranium adsorption material design. The QSAR model uses composition features and physicochemical properties to predict stable candidate materials. Given that the physicochemical properties can be calculated from the composition alone, appropriate candidate materials can be predicted prior to synthesis. This enhances the speed of candidate material discovery while reducing the cost. XGBoost achieved good performance in the test set, and AD analysis can be applied to further eliminate outlier candidates and increase the predictive performance of the model.

## Data availability

Data for this article have been included as in the ESI.†

## Author contributions

H. S. and Y. S. designed the concept of the paper together. Y. S. collected dataset and provided features for the model. H. S. analyzed dataset and trained the model. H. S. and Y. S. wrote and edited the manuscript together. H. S. prepared figures and tables.

## Conflicts of interest

The authors declare no conflict of interest.

## Supplementary Material

RA-015-D5RA02220G-s001
